# Preparation of Xanthene-TEMPO Dyads: Synthesis and Study of the Radical Enhanced Intersystem Crossing

**DOI:** 10.3390/ijms241311220

**Published:** 2023-07-07

**Authors:** Wenhui Zhu, Yanran Wu, Yiyan Zhang, Andrey A. Sukhanov, Yuqi Chu, Xue Zhang, Jianzhang Zhao, Violeta K. Voronkova

**Affiliations:** 1 State Key Laboratory of Fine Chemicals, Frontier Science Center for Smart Materials, School of Chemical Engineering, Dalian University of Technology, 2 Ling Gong Road, Dalian 116024, China; ltzhuwenhui01@mail.dlut.edu.cn (W.Z.); yanranwu@mail.dlut.edu.cn (Y.W.); 513703896@mail.dlut.edu.cn (Y.Z.); 20201013026@mail.dlut.edu.cn (Y.C.); 11907113@mail.dlut.edu.cn (X.Z.); 2 Zavoisky Physical-Technical Institute, FRC Kazan Scientific Center of Russian Academy of Sciences, Kazan 420029, Russia; ansukhanov@mail.ru

**Keywords:** electron paramagnetic resonance, intersystem crossing, photopolymerization, radical, nanosecond transient absorption spectroscopy, triplet state

## Abstract

We prepared a rhodamine-TEMPO chromophore-radical dyad (RB-TEMPO) to study the radical enhanced intersystem crossing (REISC). The visible light-harvesting chromophore rhodamine is connected with the TEMPO (a nitroxide radical) via a C–N bond. The UV-vis absorption spectrum indicates negligible electron interaction between the two units at the ground state. Interestingly, the fluorescence of the rhodamine moiety is strongly quenched in RB-TEMPO, and the fluorescence lifetime of the rhodamine moiety is shortened to 0.29 ns, from the lifetime of 3.17 ns. We attribute this quenching effect to the intramolecular electron spin–spin interaction between the nitroxide radical and the photoexcited rhodamine chromophore. Nanosecond transient absorption spectra confirm the REISC in RB-TEMPO, indicated by the detection of the rhodamine chromophore triplet excited state; the lifetime was determined as 128 ns, which is shorter than the native rhodamine triplet state lifetime (0.58 μs). The zero-field splitting (ZFS) parameters of the triplet state of the chromophore were determined with the pulsed laser excited time-resolved electron paramagnetic resonance (TREPR) spectra. RB-TEMPO was used as a photoinitiator for the photopolymerization of pentaerythritol triacrylate (PETA). These studies are useful for the design of heavy atom-free triplet photosensitizers, the study of the ISC, and the electron spin dynamics of the radical-chromophore systems upon photoexcitation.

## 1. Introduction

Triplet photosensitizers (PSs) have attracted much attention in recent years [1,2,3,4], because these compounds are significant in fundamental photochemistry studies [2,5,6], as well as in applications such as photodynamic therapy (PDT) [7,8], photocatalysis [9,10,11], photovoltaics [12], photopolymerizations [13] and triplet-triplet annihilation-based upconversion, etc. [14,15,16,17,18,19]. Triplet PSs usually show strong absorption of light, efficient intersystem crossing (ISC), appropriate excited state energy and redox potentials [2]. ISC efficiency is one of the critical factors concerning the application of the triplet PSs. ISC in organic molecules is usually enhanced by the spin-orbital coupling (SOC) effect. For instance, the heavy atom attached to a chromophore may enhance the ISC, via the so-called heavy atom effect [20], the representative compounds based on this approach are the precious metal phosphorescent complexes, such as the polyimine Ir(III), Pt(II), Ru(II), and Os(II) complexes [21,22,23,24,25], and the organic compounds containing bromine or iodine atoms in the molecular structures [2]. Organic molecules with an electronic excited state having the n−π* transition usually show efficient ISC, and the corresponding ISC complies with EI Sayed’s rule [26,27]. Recently, we and others showed that energy level matching of the S_1_ and T_n_ states (*n* > 1) may result in efficient ISC [28,29]. Although this mechanism may manifest in a twisted molecular structure, the recent evidence shows this may be just a coincidence [30,31]. Charge recombination-induced ISC is also known for organic compounds, including the radical pair ISC (RP ISC) in electron donor–acceptor dyads containing long linkers, and the recently developed spin–orbit charge transfer ISC (SOCT-ISC) in compact, orthogonal electron donor–acceptor dyads [4,32,33,34]. Fullerene, such as C_60_, has been used as an electron spin converter connected with a visible light-harvesting antenna to construct heavy atom-free triplet PSs [35,36,37,38]. Radical enhanced ISC (REISC) has also been proposed [39,40]. In this approach, a stable radical, such as the nitroxide radical (TEMPO, etc.) is covalently attached to a visible light-harvesting chromophore, and the electron spin–spin interaction between the nitroxide radical and the photoexcited chromophore will make the otherwise electron spin forbidden ISC of the light-harvesting chromophore possible [41,42]. The mechanism is that for this three-electron system, doublet (D) and quartet states (Q) are resulted due to the electron spin–spin interaction between the radical and the electronically excited chromophore. For the D_n_ state (*n* ≤ 2, etc.), populated directly upon photoexcitation into the visible light-harvesting chromophore, the chromophore is in a closed-shell electronic configuration. For the D_1_ state, however, the chromophore could be in an open-shell electronic configuration (i.e., the chromophore has a total spin angular momentum of S^2^ = 2). D_n_ → D_1_ transition is overall electron spin allowed; thus, the ISC of the chromophore becomes spin allowed. As a result, attachment of a stable radical to a chromophore usually results in the ISC of the light-harvesting chromophore [39,40,43]. Some molecular systems based on this approach have been reported, such as those based on chromophores of Bodipy [39,44], perylenebisimide (PBI) [42,45], naphthalenediimide (NDI) [40], and cyanine dyes [43], etc.

However, it is clear that much room is left for using this molecular structure protocol to design new heavy atom-free triplet PSs. For instance, the selection of a proper chromophore and the linker between the radical and the chromophore is critical to optimize the photophysical property of the resulted dyad for the purpose of designing an ideal triplet PSs [39]. Moreover, the triplet state lifetimes are strongly dependent on the structure of the chromophore and the linker (length, π-conjugation, or saturated structure, etc.) [39,46]. More chromophores are needed to elucidate the relation between the molecular structure and the triplet state property. We have noticed that the triplet state lifetime of a specific chromophore is dependent on the ISC mechanism. Rhodamine is a popular chromophore showing strong absorption of visible light, and has been widely used in the study of fluorescence, molecular probes, and luminescence bioimaging. However, to our surprise, this chromophore has not been used for REISC.

**Scheme 1:**
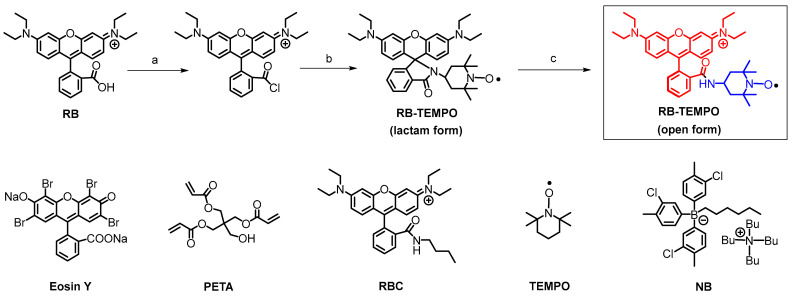
Synthesis of the Compounds. Key: (a) Rhodamine B, phosphorus oxychloride, dry DCM, 45 °C, reflux 5 h under N_2_, directly used in the next reaction. (b) 4-Amino-TEMPO, dry triethylamine, dry acetonitrile, 80 °C, reflux 21 h under N_2_, directly used in the next reaction. Yield: 10%. (c) trifluoroacetic acid.

In order to address the above challenges, herein we prepared a rhodamine-TEMPO (RB-TEMPO, Scheme 1); the photophysical property, especially the ISC and the triplet state property, was studied by using steady state UV-vis absorption spectra, fluorescence spectra, and nanosecond transient absorption spectra. A preliminary study on the photopolymerization with RB-TEMPO as a photoinitiator is also presented.

## 2. Results and Discussion

### 2.1. Molecular Structure Design Rationales

We prepared RB-TEMPO (Scheme 1), which is feasibly synthesized with the commercially available rhodamine and the 4-amino-2,2,6,6-tetramethylpiperidine-1-oxyl (TEMPO) [47,48]. This compound was reported previously [49,50], However, it was used as fluorescent molecular robes, the photophysical property was not studied in detail. For the selective detection of biologically related radicals, the ISC and the triplet excited state were not studied. The yield is satisfactory; the molecular structure is verified by ^1^H NMR and HRMS (see Figures S1 and S2). Compounds used as reference compounds in the studies are also presented in Scheme 1.

### 2.2. UV-Vis Absorption and Fluorescence Spectra

**Figure 1:**
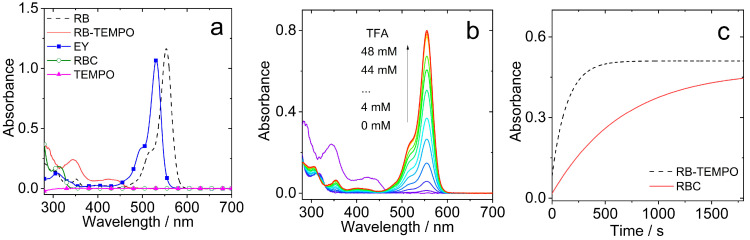
(**a**) UV–vis absorption spectra of the compounds in ethanol (EtOH); (**b**) UV-Vis absorption spectra change with the addition of trifluoroacetic acid added to RB-TEMPO solution in EtOH; (**c**) Kinetics of the lactam/opened amide transformation of the Rho moiety for these compounds, monitored at 555 nm upon addition of TFA (96 mM) in EtOH. *c* = 1.0 × 10^−5^ M, 20 °C.

The UV-vis absorption spectra of the compounds were studied (Figure 1 and Figures S3–S5). RB shows the characteristic strong absorption band at 554 nm (ε = 1.16 × 10^5^ M^−1^ cm^−1^). For RB-TEMPO, however, no such absorption band was observed, indicating that the RB-TEMPO molecules adopt a closed ring, lactam structure. TEMPO does not show any strong absorption in the visible spectral range. In order to confirm that RB-TEMPO has a lactam structure, we studied the evolution of the UV-vis absorption spectra of RB-TEMPO in the presence of acid in a protic solvent (EtOH. Figure 1b). With strong acid, trifluoroacetic acid (TFA), added, a strong absorption band at 555 nm developed (Figure 1b); and changes in absorbance over time are also studied (Figure S8); this absorption band is virtually the same as that of the native rhodamine (Figure 1a) and this process is reversible. This phenomenon was also observed in dichloromethane (DCM. Figure S7) and acetonitrile (ACN. Figure S11). With a base added, the molecular structure changes to the closed form (Figure S9). We also tested the UV–vis absorption spectrum of RB-TEMPO with the addition of trifluoroacetic acid in dichloromethane, acetonitrile, and methanol; similar results were observed (Figures S6, S10 and S12).

We also studied the kinetics of the transformation of RB-TEMPO from the lactam structure to the opened amide structure, by following the absorbance at 555 nm (Figure 1c). RBC is a common rhodamine compound, and its ring-opening kinetics have been reported [51]. The alkyl chain has little effect on the photophysical properties of its parent material, so it was selected as a reference compound to study the effect of connecting the TEMPO on the ring-opening kinetics. Interestingly, we found RB-TEMPO has faster kinetics (*k* = 7.1 × 10^−3^ s^−1^ under the experimental condition) than the rhodamine reference compound RBC (*k* = 1.5 × 10^−3^ s^−1^). The UV-Vis absorption spectra of RBC with the addition of TFA is shown in Figure S13. This information is useful for subsequent studies, because in order to ensure a complete lactam → opened amide structure transformation, the appropriate experimental condition has to be applied, such as the solvent, the acid, and the waiting time after the addition of acid in the solution.

**Figure 2:**
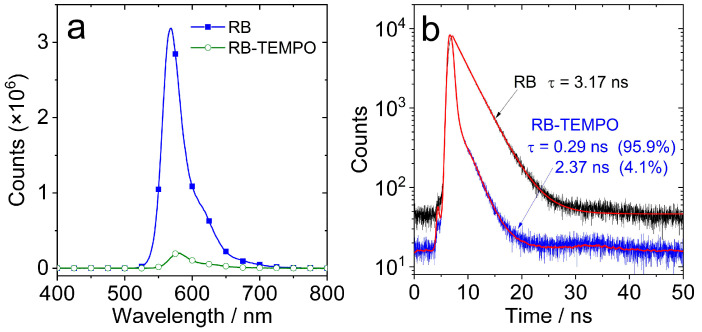
(**a**) Fluorescence emission spectra of RB and RB-TEMPO in EtOH with TFA (48 mM) added. Optically matched solutions were used in each panel (each of the solutions gives the same absorbance at the excitation wavelength: *λ*_ex_ = 300 nm, *A*_300nm_ = 0.101); (**b**) the corresponding fluorescence decay traces of RB and RB-TEMPO with TFA (48 mM) at 570 nm in EtOH. The black and blue lines are the measured curves, and the red lines are the fitted curves. *λ*_ex_ = 510 nm. *c* = 1.0 × 10^−5^ M, 20 °C.

The fluorescence property of the compounds was studied (Figure 2). In Figure 1a, we can observe that RB and RB-TEMPO indicate absorption at 300 nm, so we excited these two compounds at 300 nm. RB gives a strong fluorescence band centered at 570 nm, under an acid condition in a polar solvent. In contrast, RB-TEMPO gives much weaker fluorescence, at the similar wavelength (Figure 2a). The quenching of the fluorescence of RB is attributed to the electron spin–spin interaction of the radical with the photoexcited RB chromophore; it is unlikely due to the photo-induced electron transfer or Förster singlet energy transfer [52]. Although the quenching of fluorescence of RB by attaching TEMPO was reported previously, and used as a fluorescence switch-on molecular probe for the detection of reactive radicals such as CH_3_^•^, the quenching mechanism was not studied in detail [49,50]. The fluorescence spectra of EY and RBC are given in supplementary information (Figures S14 and S15).

The fluorescence lifetimes of the compounds were studied (Figure 2b). For the reference compound RB, the fluorescence lifetime was determined as 3.17 ns (monoexponential decay). For RB-TEMPO, however, a much shorter fluorescence lifetime was observed as 0.29 ns (95.9%)/2.37 ns (4.1%). The faster relaxation of the emissive S_1_ state of the rhodamine moiety in RB-TEMPO is attributed to the REISC effect (see later section).

**Figure 3:**
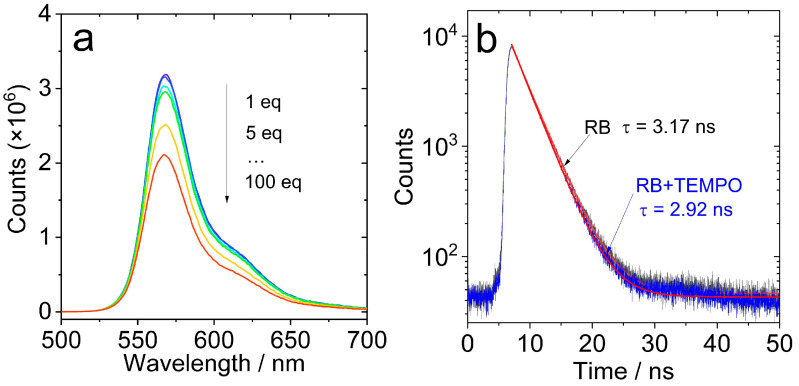
(**a**) Fluorescence emission spectra change with incremental amount of TEMPO added to RB solution in EtOH. Optically matched solutions were used in each panel (each of the solutions gives the same absorbance at the excitation wavelength: *λ*_ex_ = 300 nm, *A*_300nm_ = 0.101) and 20 °C; (**b**) the corresponding fluorescence decay traces of RB and RB with 50 eq. of TEMPO at 570 nm in EtOH. The blue line is the measured curve, and the red line is the fitted curve. *λ*_ex_ = 510 nm. *c* = 1.0 × 10^−5^ M, 20 °C.

In order to rule out the possibility that the fluorescence quenching in RB-TEMPO is caused by any intermolecular interaction, we also studied the fluorescence of compound RB in the presence of TEMPO (Figure 3). The results show that the fluorescence of RB was quenched to a minor extent, even in the presence of a large excess of TEMPO (100 equiv. Figure 3a). The fluorescence lifetime study shows that the fluorescence lifetime is 2.92 ns for the mixture of RB and TEMPO (50 equiv.). These results show that the quenching of the fluorescence of RB by intermolecular interaction is much weaker than the intramolecular quenching effect in RB-TEMPO. This is reasonable because the fluorescence lifetime of the rhodamine is short (a few ns); in comparison, the molecular diffusion is a much slower process, and the majority of molecules at the S_1_ state emit fluorescence before encountering with the TEMPO radical via diffusion in the fluid solution. We also measure the fluorescence lifetime of EY (Figure S16).

The photophysical parameters of the compounds are presented in Table 1. It was found the fluorescence quantum yield of RB-TEMPO (*Φ*_F_ = 26.9%) is much lower than RB (*Φ*_F_ = 62.7%). As an approximation of the ISC quantum yields, we measured the singlet oxygen (^1^O_2_) quantum yields (*Φ*_Δ_) of the compounds. RB has no ^1^O_2_ photosensitizing ability, and the *Φ*_Δ_ is null. For RB-TEMPO, however, the *Φ*_Δ_ was determined as 40.1%. This is attributed to the REISC effect, due to the electron spin–spin interaction between the TEMPO and the photoexcited RB moiety. The raw data for singlet oxygen yield are listed in the Table S1.

### 2.3. Nanosecond Transient Absorption (ns-TA) Spectroscopy: Properties of the Triplet States

**Figure 4:**
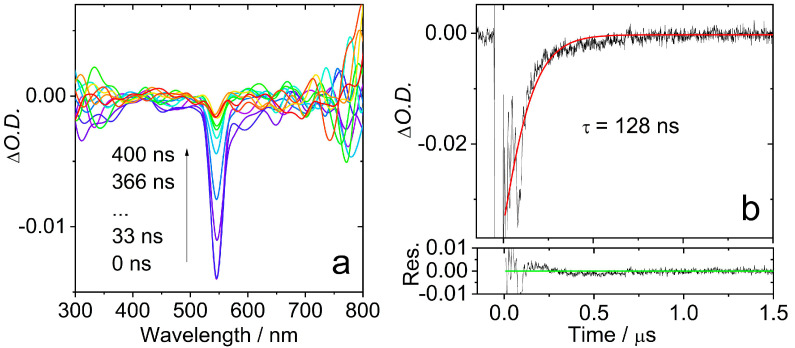
(**a**) Nanosecond transient absorption spectra of RB−TEMPO upon pulsed laser excitation, the direction of the arrow indicates the increase of the delay time. (*λ*_ex_ = 550 nm) and (**b**) the corresponding decay trace of RB-TEMPO monitored at 545 nm, (The red line is obtained by fitting the black decay curve), *c* = 1.0 × 10^–5^ M, in de-aerated methanol, 20 °C.

In order to study the formation of the triplet state of RB moiety in RB-TEMPO, the ns-TA spectra upon pulsed laser photoexcitation of the compound were recorded (Figure 4). A ground state bleaching band (GSB) centered at 545 nm was observed (Figure 4a), but no excited stated absorption (ESA) band was observed, due to the weak signal-to-noise ratio.

The transient species lifetime was determined as 128 ns (Figure 4b), by monitoring the decay trace at 545 nm. This short lifetime is reasonable, because the nitroxide radical center is close to the RB chromophore; this character may lead to strong electron spin–spin interaction between the radical and the photoexcited chromophore. Consequently, it may shorten the triplet state lifetime [39]. Previously, the triplet state lifetime of the native rhodamine B was determined as 0.58 μs (in methanol) [55]. It is reported that the acridine triplet state can be used to sensitize the formation of rhodamine triplet states. It is unlikely that the direct excitation of rhodamine would lead to observable transient excited states on the microsecond timescales, since the quantum yield for an intersystem crossing is too low. The triplet state lifetime of RB was also measured by direct excitation, as 94 ns (Figure S17).

This result shows that the linker length (and also the electronic feature) should be optimized for designing a heavy atom-free triplet PSs based on the REISC mechanism. Previously, a short triplet state lifetime of perylene was determined as 100 ns in a perylene–nitroxide dyad, which is attributed to the short distance between the nitroxide spin carrier and the perylene chromophore. We show that in Bodipy–TEMPO dyads, the longer the linker is, the longer the triplet state lifetime of the Bodipy moiety may have. However, the ISC quantum yield may be low for dyads containing a long linker [39].

It should be pointed out that here the ‘triplet’ state refers to the RB chromophore’s electronic configuration. Overall, the photoexcited state of RB-TEMPO is a three-electron spin system; only the doublet (square of spin angular momentum S^2^ = 3/4) and quartet state (square of angular momentum S^2^ = 15/4) exist. However, the optical spectroscopy, such as the ns-TA spectroscopy, is unable to discriminate the doublet state and the quarter state, because these states share similar energy, and the difference is the electron spin–spin interaction. Moreover, the absorption of the D_1_ and Q_1_ state are virtually the same, i.e., the electronic transition to higher states of the chromophore or the TEMPO radical. This scenario is different from the S_1_ state and T_1_ state of the same chromophore. In a two-electron spin system, in this case, drastically different excited state absorption spectra can be found for the S_1_ and T_1_ states.

**Figure 5:**
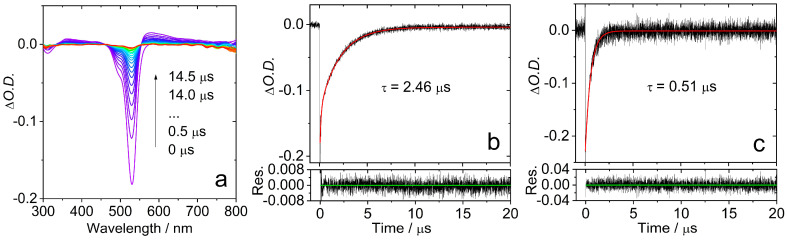
(**a**) Nanosecond time-resolved transient absorption spectra of Eosin Y upon pulsed laser excitation, the direction of the arrow indicates the increase of the delay time. (*λ*_ex_ = 520 nm). (**b**) Decay trace of Eosin Y at 525 nm, *c* = 1.0 × 10^–5^ M, in de-aerated methanol. (**c**) Triplet state lifetime after TEMPO quenching of Eosin Y at 525 nm (TEMPO radical quenched triplet lifetime), the red lines are obtained by exponential fitting the black decay curve, *c* [EY] = 1.0 × 10^–5^ M, *c* [TEMPO] = 5.0 × 10^–4^ M in de-aerated methanol, 20 °C.

As a control experiment, we measured the ns-TA spectra of Eosin Y (Figure 5). Due to the presence of four bromine atoms attached to the chromophore, the ISC should be enhanced by the heavy atom effect. A GSB band centered at 530 nm was observed (Figure 5a), together with a weak ESA band in the range of 300–500 nm and 550–800 nm. The triplet state lifetime was determined as 2.46 μs at 525 nm (Figure 5b) and 3.25 μs at 600 nm (Figure S19).The triplet state lifetime was determined as 2.46 μs (Figure 5b). Previously, the triplet state lifetime of Eosin Y was determined as 0.6 μs in an aerated solution [56]. Eosin Y absorbed on a solid matrix may show a much longer triplet state lifetime [57].

We also studied the change of the triplet state lifetime of Eosin Y in the presence of TEMPO (Figure 5c). We found that the triplet state lifetime of Eosin Y was shortened to 0.51 μs in the presence of 50 eq. of TEMPO in the solution. The results with 5 eq., 10 eq., 20 eq. and 30 eq. of TEMPO are added into Eosin Y solution are also shown (Figure S18).The quenching should be via the radical triplet pair mechanism, which is controlled by molecular diffusion. Given the TEMPO is attached to the RB via a short linker, the triplet state lifetime of the RB moiety should be significantly shortened; we propose this is the case for RB-TEMPO.

### 2.4. Pulsed Laser Excited Time-Resolved Electron Paramagnetic Resonance (TREPR) Studies

It is known that the electron spin dynamics of the radical-light-harvesting chromophore dyad, a three-electron spin system, can be studied with TREPR spectra [41,58]; usually a quartet state can be observed [59,60,61]. We do not observe an intense TREPR spectrum for RB-TEMPO that could be confidently attributed to the interaction of the triplet state with the radical.

**Figure 6:**
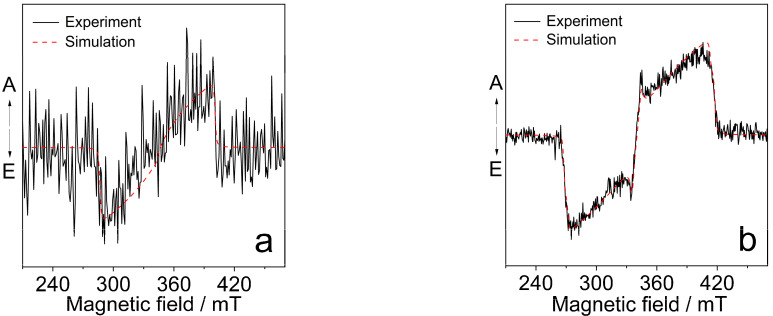
Experimental and simulation TR EPR spectra of (**a**) RB and (**b**) Eosin Y. Simulation parameters are presented in the table below. Excited with nanosecond pulsed laser at 525 nm with energy 1 mJ. *c* = 1.0 × 10^−4^ M in ethanol, in frozen solution at 80 K.

We noted that the zero-field splitting (ZFS) *D* and *E* parameters of xanthene dyes were rarely reported previously [62]. Therefore, we measured the TREPR spectrum of rhodamine B (RB. Figure 6a). Because of the poor ISC of RB, the TREPR spectrum is noisy. A simulation of the TREPR spectrum gives the ZFS *D* and *E* parameters as −1600 MHz (0.0534 cm^−1^) and 530 MHz (0.0177 cm^−1^) (Table 2), respectively. This is similar to the previous report (*D* = 0.058 cm^−1^, *E* = 0.017 cm^−1^) [62,63]. Previously, the interaction between the rhodamine and radical was studied, however, the ZFS parameters were not reported [64].

Since the ISC of RB is poor, we studied another xanthene dye that shows efficient ISC, i.e., Eosin Y (Figure 6b). As compared to Figure 6a, the signal of Figure 6b is stronger. We propose it is probably due to the longer triplet state lifetime of EY as compared to that of RB, and the higher singlet oxygen (^1^O_2_) quantum yield (can be approximated as the ISC yield) than RB; all these factors contribute to the better signal for EY. Upon a pulsed laser excitation, an electron spin polarization (ESP) pattern of Eosin Y was observed. This is the typical feature of the TREPR spectrum of a triplet state produced by the spin–orbit coupling effect. With spectral simulation, the ZFS *D* and *E* parameters were determined as −2100 MHz and 618 MHz (Table 2). This is close to the previously reported 2428 MHz for the *D* parameter (the *E* parameter was not reported) [62]. In the future, we will design new radical–rhodamine dyads to observe the quartet state.

### 2.5. Photopolymerization and Photobleaching

**Figure 7:**
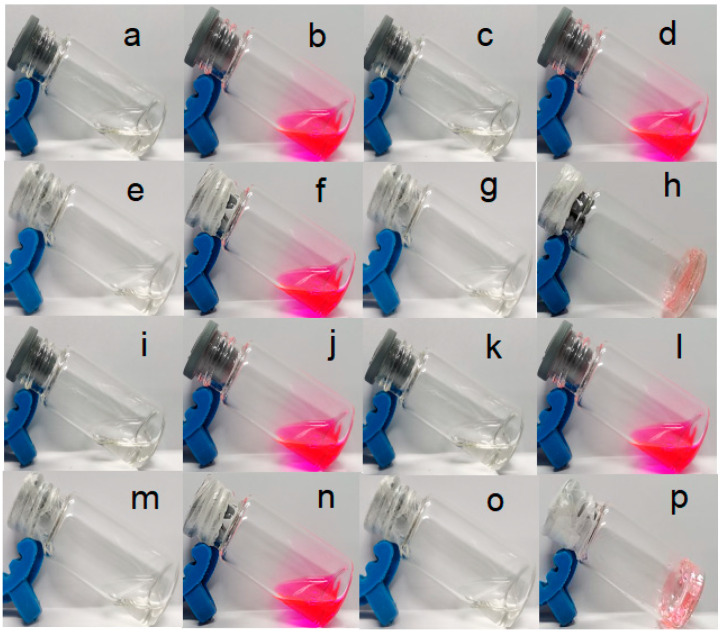
The photopolymerization of the monomer PETA under N_2_, upon photoirradiation using a 35 W Xenon lamp (unfiltered white light intensity: 80 mW/cm^2^, light dose: 4.8 × 10^3^ mJ/cm^2^) with different photoinitiators and co-initiators. The first two rows are RB-TEMPO photopolymerization experiments for the solution of (**a**)/(**e**) PETA alone, (**b**)/(**f**) PETA/RB-TEMPO, (**c**)/(**g**) PETA/NB, (**d**)/(**h**) PETA/RB-TEMPO/NB. The first row is before the photoirradiation and the second row is after the photoirradiation. The third and fourth rows are the photopolymerization experiments with the **RB** as the photoinitiator under similar conditions. (**i**)/(**m**) PETA alone, (**j**)/(**n**) PETA/RB, (**k**)/(**o**) PETA/NB, (**l**)/(**p**) PETA/RB/NB. The third row are the samples before the photoirradiation and the fourth row is after the photoirradiation. Photosensitizer: 6 wt%. The photopolymerization activity is shown in the gelation of liquid blends upon photoirradiation.

In order to study the potential application of the compounds, such as in photopolymerization, we studied the photopolymerization of pentaerythritol triacrylate (PETA), with RB-TEMPO as a photoinitiator (Figure 7) [65,66,67]. We selected tetrabutylammonium tris(3-chloro-4-methylphenyl)hexylborate (NB) as the co-initiator, which is known to produce highly reactive hexyl radicals to initiate radical polymerization upon giving an electron to the photoexcited triplet PSs. The results show that the PETA is polymerized upon photoirradiation of the mixture of RB-TEMPO/NB/PETA (Figure 7h), and photoirradiation of the mixture in the absence of RB does not lead to photopolymerization, indicated by the solidification of the fluid mixture.

**Scheme 2:**
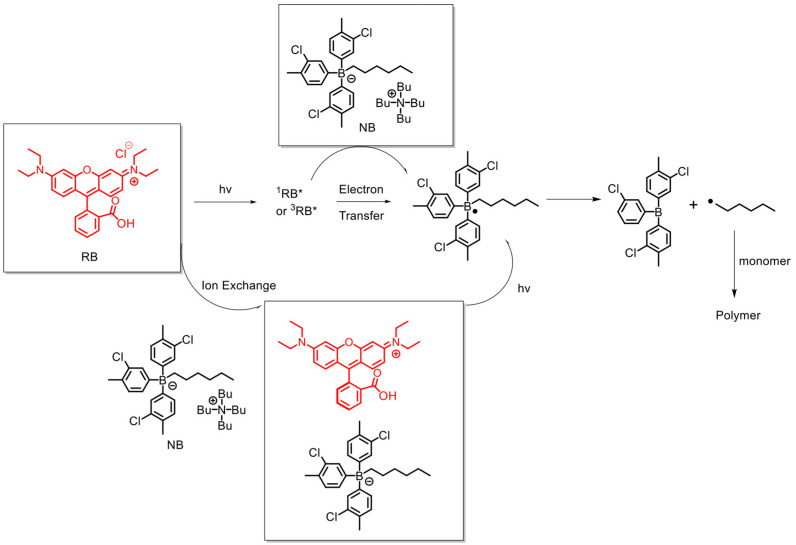
Photochemical mechanisms of a generation of radicals for the RB/NB system. Note for RB-TEMPO, the ion exchange reaction may exist as well; therefore intramolecular electron transfer may occur, which leads to the generation of the hexyl radical and photopolymerization results as well. (“*” represents the excited state of the photosensitizer, “1” represents the singlet state, “3” represents the triplet state.)

The photopolymerization of PETA in the presence of RB-TEMPO and NB is presented in Scheme 2. We propose there is electron transfer from NB to the photoexcited RB-TEMPO; the hexyl radical initiates the polymerization of the PETA monomer.

Interestingly, we found that the reference compound RB is also active to initiate the photopolymerization of PETA (Figure 7p). Note the fluorescence lifetime of RB is short (3.17 ns), considering the ionic character of both RB and NB. We proposed there exists an ion exchange reaction between RB and NB. The boronate anion may become the counter anion of the RB molecules; as a result, intramolecular electron transfer between the boronate anion and the xanthene cation of RB is possible, which produces the hexyl radical and subsequently it initiates the polymerization of PETA. This is supported by the change of the UV-Vis absorption spectra of RB after mixing with NB (Figure S21 in the Supplementary Information) [68].

In order to study the intermolecular electron transfer or intramolecular electron transfer, after the ion exchange upon mixing of RB/RB-TEMPO (with NB), the UV-vis absorption spectra of RB and RB-TEMPO in the presence of NB upon photoirradiation were recorded (Figure S21 in the Supplementary Information). Upon photoirradiation of the mixture, the absorption of the RB chromophore centered at 555 nm is diminished, indicating the photobleaching of the rhodamine chromophore, as a result of inter/intramolecular electron transfer (Figure S21 in the Supplementary Information). And the fluorescence lifetime of RB in the presence of NB was also measured (Figure S24). As a comparative experiment, we also detect the UV-Vis absorption spectrum of RB only and find no significant changes in absorbance (Figure S22) which proves the light stability of RB. Under the dark condition, the absorbance change after adding NB to RB is not obvious, indicating that the electron transfer process can only occur after RB is excited by light (Figure S23). Interestingly, we found the photobleaching is more significant for RB, than RB-TEMPO (Figure S20 in the Supplementary Information). Considering that the ion exchange reaction may occur, intramolecular electron transfer is possible. Note the singlet state lifetime of RB (3.17 ns) is longer than RB-TEMPO (0.29 ns); thus, the more significant photobleaching for RB/NB is reasonable. It should be pointed out that the triplet excited state of the rhodamine moiety is unable to drive an electron transfer with NB as the electron donor. We obtained the electron transfer free energy change through theoretical calculation, and the original data are listed in the Table S2.

## 3. Materials and Methods

### 3.1. General Methods and Material

All the chemicals used for the synthesis were analytically pure and were used as received without further purification. UV-vis absorption spectra were obtained on a UV-2550 spectrophotometer (Shimadzu Ltd., Kyoto, Japan). Fluorescence spectra were recorded on an FS5 spectrofluorometer (Edinburgh Instrument Ltd., Livingston, UK). Fluorescence quantum yields (*Φ*_F_) were measured with an absolute photoluminescence quantum yield spectrometer (Quantaurus-QY plus C13534-11, Hamamatsu Ltd., Hamamatsu, Japan). Fluorescence lifetime was measured with an OB920 luminescence lifetime spectrometer (Edinburgh Instruments Ltd., Livingston, UK). The synthetic procedure and molecular structural characterization data of the compound, i.e., RB-TEMPO, are collected in the following sections. All compounds used in the experiments and presented in Scheme 1 were purchased.

### 3.2. Compound RB-TEMPO

Rhodamine B (750 mg, 1.7 mmol) was dissolved in dry dichloromethane (50 mL), and then phosphorus oxychloride (1 mL) was added under a N_2_ atmosphere. The reaction mixture was refluxed with stirring under a N_2_ atmosphere for 5 h. Then, the reaction mixture was cooled to room temperature. The solvent is removed under reduced pressure. Then, the residue is dissolved in dry acetonitrile (80 mL). 4-amino-TEMPO (344 mg, 2 mmol) and triethylamine (2 mL) were added under a N_2_ atmosphere. After stirring at room temperature for 15 min, the reaction solution was refluxed for 21 h. After the reaction is finished, the reaction mixture is cooled to room temperature. The solvent was removed under reduced pressure. The crude product was purified by column chromatography (silica gel; dichloromethane/methanol, 25:1, *v*/*v*) to afford a red solid in 10% yield (0.101 g). The ^1^H NMR spectrum was recorded after treatment of the product with phenylhydrazine, to reduce the nitroxide radical to a hydroxyl structure (no satisfactory NMR spectra can be recorded for the paramagnetic compound). ^1^H NMR (400 MHz, CDCl_3_): *δ* 0.82–0.89 (m, 2 H), 1.03 (s, 5 H), 1.16 (t, *J* = 6.88 Hz, 12 H), 1.39 (s, 5 H), 1.80 (s, 2 H), 2.88 (s, 2 H) 3.35 (s, 8 H), 6.42 (s, 6 H), 6.87–6.93 (m, 1 H), 7.16 (s, 2 H), 7.53 (s, 2 H), 7.92 (s, 1 H). HRMS (EI): calcd for C_37_H_48_N_4_O_3_^+·^, *m*/*z* 596.3721; found *m*/*z* 596.3711.

### 3.3. Nanosecond Transient Absorption (ns TA) Spectroscopy

The ns TA spectra were measured on LP980 laser flash photolysis spectrometers (Edinburgh Instruments, Livingston, UK). Samples were purged with N_2_ for 15 min before measurements, and excited with a nanosecond pulsed laser (Quantel Nd:YAG nanosecond pulsed laser for LP980, OpoletteTM 355II+UV laser for LP980). The signal was digitized with a Tektronix TDS 3012B oscilloscope and the data were processed with L900 software. (https://www.edinst.com/products/l900-software/, access on 30 May 2023).

### 3.4. Time-Resolved Electron Paramagnetic Resonance (TREPR) Spectra

Samples were dissolved in ethanol. O_2_ was removed by a few freeze–pump–thaw cycles. The time-resolved continuous-wave (TR CW) EPR spectral measurements were performed on an X-band EPR Elexsys E-580 spectrometer (Bruker, Saarbrucken, Germany) at 80 K. Samples were excited by laser pulse at 525 nm with 1 mJ energy per pulse. The data were processed with the EasySpin package based on Matlab [69].

### 3.5. Photopolymerization and Photobleaching Experiments

#### 3.5.1. Photopolymerization

In the experiment, four control experiments were carried out. Monomer PETA (1.2 g); PETA (1.2 g) and photosensitizer (0.07 mg); PETA (1.2 g) and NB (2 mg); PETA (1.2 g), NB (2 mg) and photosensitizer (0.07 mg) were added into de-aerated acetonitrile (0.1 mL), respectively. Then, the mixture was photoirradiated under the unfiltered white light of a xenon lamp (light intensity: 80 mW/cm^2^), and the polymerization time was recorded to compare the rate of polymerization.

#### 3.5.2. Photobleaching Experiments

In the experiments, a set of comparison experiments was carried out. In the first experiment, the RB (*c* = 1.0 × 10^–5^ M) was added into de-aerated acetonitrile (3 mL) to measure its UV-vis absorption spectrum. Then, the co-initiator NB (2 mg) was added to the RB solution. After each 1 min of xenon lamp photoirradiation (unfiltered white light intensity: 80 mW/cm^2^), the absorption spectra were measured to monitor the photobleaching in the presence of co-initiator NB. The UV-vis absorption spectra of RB solution in the absence of NB were also photoirradiated by a xenon lamp (unfiltered white light intensity: 80 mW/cm^2^) and was also measured to study the photo-stability.

### 3.6. Density Functional Theory (DFT) Calculations

DFT calculations were performed by using the Gaussian 09 package [70]. The ground state geometries and orbital energies were optimized using DFT with the B3LYP functional and the 6-31G (d) basis set. The excitation energy of the compounds was calculated by TD-DFT at the B3LYP/6-31G (d) level.

## 4. Conclusions

In summary, in order to study the radical enhanced intersystem crossing (REISC), we prepared a rhodamine-TEMPO chromophore–radical dyad (RB-TEMPO). The visible light-harvesting chromophore rhodamine is connected with the TEMPO (nitroxide radical) via the C–N bond. The UV-vis absorption spectrum indicates a negligible electron interaction between the two units at the ground state. Interestingly, fluorescence of the rhodamine moiety is strongly quenched in RB-TEMPO, and the fluorescence lifetime of the rhodamine moiety is shortened to 0.29 ns, from that of 3.17 ns of the native rhodamine chromophore. We attribute this quenching effect to the intramolecular electron spin–spin interaction between the nitroxide radical and the photoexcited rhodamine chromophore. This conclusion is supported by the control experiment which shows the intermolecular interaction of the rhodamine and TEMPO leads to a much weaker quenching effect. Nanosecond transient absorption spectra confirm the REISC effect in RB-TEMPO, indicated by the detection of the rhodamine chromophore triplet excited state; the triplet state lifetime was determined as 0.128 ns, which is shorter than the rhodamine triplet state lifetime (0.58 μs). The zero-field splitting (ZFS) parameters of the triplet state of the chromophore were determined with the pulsed laser excited time-resolved electron paramagnetic resonance (TREPR) spectra as *D* = −1600 MHz and *E* = 530 MHz. RB-TEMPO was used as a photoinitiator for the photopolymerization of pentaerythritol triacrylate (PETA), in the presence of a co-initiator (also acts as an electron donor) tetrabutylammonium tris(3-chloro-4-methylphenyl)hexylborate. Intermolecular and intramolecular (ion exchange reaction exist) electron transfer between the rhodamine chromophore and the co-initiator exist, leading to the production of the alkyl radical, which initiates the radical polymerization of the monomers. These studies are useful for the design of heavy atom-free triplet photosensitizers and study of the ISC and electron spin dynamics of these versatile organic molecules.

## Figures and Tables

**Table 1:** Photophysical Data of the Compounds *^a^*.

Compounds	*λ*_abs_ ^*a*^/nm *^b^*	*ε ^c^*	*λ*_em_/nm *^d^*	*Φ*_F_/% *^e^*	*τ*_F_/ns *^f^*	*τ*_T_/ns *^g^*	*Φ*_Δ_/% *^h^*
RB	554 531 *^m^*	11.55	570 560 *^m^*	62.7	3.17 *^i^* 5.97 *^m^*	94	0 *^k^*
EY	531	10.70	548	55.3	0.95 *^j^* (6.9%)/ 3.13 *^j^* (93.1%)	2460	23.0 *^l^*
RB-TEMPO (Open Form)	555	7.99	570	26.9	0.29 *^i^* (95.9%)/ 2.37 *^i^* (4.1%)	128	40.1 *^l^*

*^a^* In EtOH (*c* = 1.0 × 10^−5^ M). *^b^* UV-Vis absorption maximum. *^c^* Molar absorption coefficient, *ε*: 10^4^ M^−1^ cm^−1^. *^d^* Fluorescence emission maximum. *^e^* Absolute fluorescence quantum yields in EtOH, determined with optical integration sphere. *^f^* Fluorescence lifetime, *λ*_ex_ = 510 nm. The numbers in parentheses show the proportions of the components. *^g^* Measured by nanosecond transient absorption. *^h^* Singlet oxygen quantum yield in EtOH. Methylene Blue (*Φ*_Δ_ = 0.57 in DCM) was used as standard [53]. *^i^ λ*_em_ = 570 nm. *^j^ λ*_em_ = 548 nm. *^k^* Not observed. *^l^ λ*_ex_ = 540 nm. *^m^* These values are cited from this reference [54].

**Table 2:** Experimental ZFS parameter, population rates of the three sublevels of the triplet states, and g-factor of the samples.

Sample	*D*/MHz	*E*/MHz	*T_x_*	*T_y_*	*T_z_*	*g*-Factor
RB	−1600	530	0	0	1	2.005
Eosin Y	−2100	618	0	0	1	2.005

## Data Availability

Original data can be requested from the corresponding author.

## Supplementary Materials

The following supporting information can be downloaded at: https://www.mdpi.com/article/10.3390/ijms241311220/s1. Synthesis of compounds, molecular structural characterization and additional spectra of compounds. Reference [71] is cited in the supplementary materials.
