# Supplementary material for: Preparation of Xanthene-TEMPO Dyads: Synthesis and Study of the Radical Enhanced Intersystem Crossing
Source: Int J Mol Sci. 2023 Jul 7;24(13):11220. doi: 10.3390/ijms241311220 (PMC10342664; doi:10.3390/ijms241311220)
Supplement: Supplementary file 1 [file ijms-24-11220-s001.zip › ijms-2451640-supplementary.pdf]

Supporting Information for:

Preparation of Xanthene-TEMPO dyads: Synthesis and Study of the Radical Enhanced Intersystem Crossing

Wenhui Zhu,[a‡] Yanran Wu,[a‡] Yiyan Zhang,[a] Andrey A. Sukhanov,[b] Yuqi Chu,[a] Xue Zhang,[a] Jianzhang Zhao[a,*] and Violeta K. Voronkova [b,*]

[a] State Key Laboratory of Fine Chemicals, Frontier Science Center for Smart Materials, School of Chemical Engineering, Dalian University of Technology, 2 Ling Gong Road, Dalian 116024, P. R. China. *E-mail: zhaojzh@dlut.edu.cn

[b] Zavoisky Physical-Technical Institute, FRC Kazan Scientific Center of Russian Academy of Sciences, Kazan 420029, Russia. E-mail: vor18@yandex.ru

.

‡ These authors contributed equally to this work.

Contents

1. Syntheses of Compounds ············ S3
2. NMR and HRMS Spectra ············ S4
3. UV–vis Absorption Spectra ············ S5
4. Fluorescence Spectra ············ S11
5. Fluorescence Lifetime Spectra ············ S12
6. Singlet Oxygen Quantum Yields ············ S12
7. Nanosecond Transient Absorption Spectra ············ S13
8. Photopolymerization and photobleaching ············ S15
9. Electrochemical Study ············ S17
10. Reference ············ S17

1. Syntheses of Compounds

Synthesis of the compound **1.** Rhodamine B（750 mg, 1.7 mmol）was dissolved in dry dichloromethane(50 mL), and then phosphorus oxychloride (1 mL) was added under nitrogen condition. The reaction liquid was stirred under nitrogen condition for reflux for 5 hours. When the temperature of the reaction liquid drops to room temperature, it is transferred to a flask. The excess solvent in the reaction mixture is removed under reduced pressure. Then the remaining residual is dissolved in dry acetonitrile (80 mL). 4-amino-TEMPO (344 mg, 2 mmol) and triethylamine (2 mL) were added under N_2 condition, after stirring at room temperature for 15 min., the reaction solution was refluxed for 21 h. After the reaction, the reaction mixture was cooled to room temperature. The excess solvent was removed under reduced pressure. The crude product was purified by column chromatography (silica gel; dichloromethane/methanol, 25:1, v/v) to afford red solid in 10% yield (0.10 g). The low yield may be due to high steric hindrance about the linkage in the molecular structure. We didn't check what the by-products were, however, we have monitored the reaction process through TLC analysis, and most of the 'side products' are unreacted starting materials. ^{1}H NMR (400 MHz, CHLOROFORM-d) ^{1}H NMR (400 MHz, $CDCl_3$): δ 0.82–0.89 (m, 2 H), 1.03 (s, 5 H), 1.16 (t, J = 6.88 Hz, 12 H), 1.39 (s, 5 H), 1.80 (s, 2 H), 2.88 (s, 2 H), 3.35 (s, 8 H), 6.42 (s, 6 H), 6.87–6.93 (m, 1 H), 7.16 (s, 2 H), 7.53 (s, 2 H), 7.92 (s, 1 H). HRMS (EI): calcd for $C_{37}H_{48}N_4O_3^+$, *m/z* 596.3721; found *m/z* 596.3711.

2. NMR and HRMS Spectra of compounds

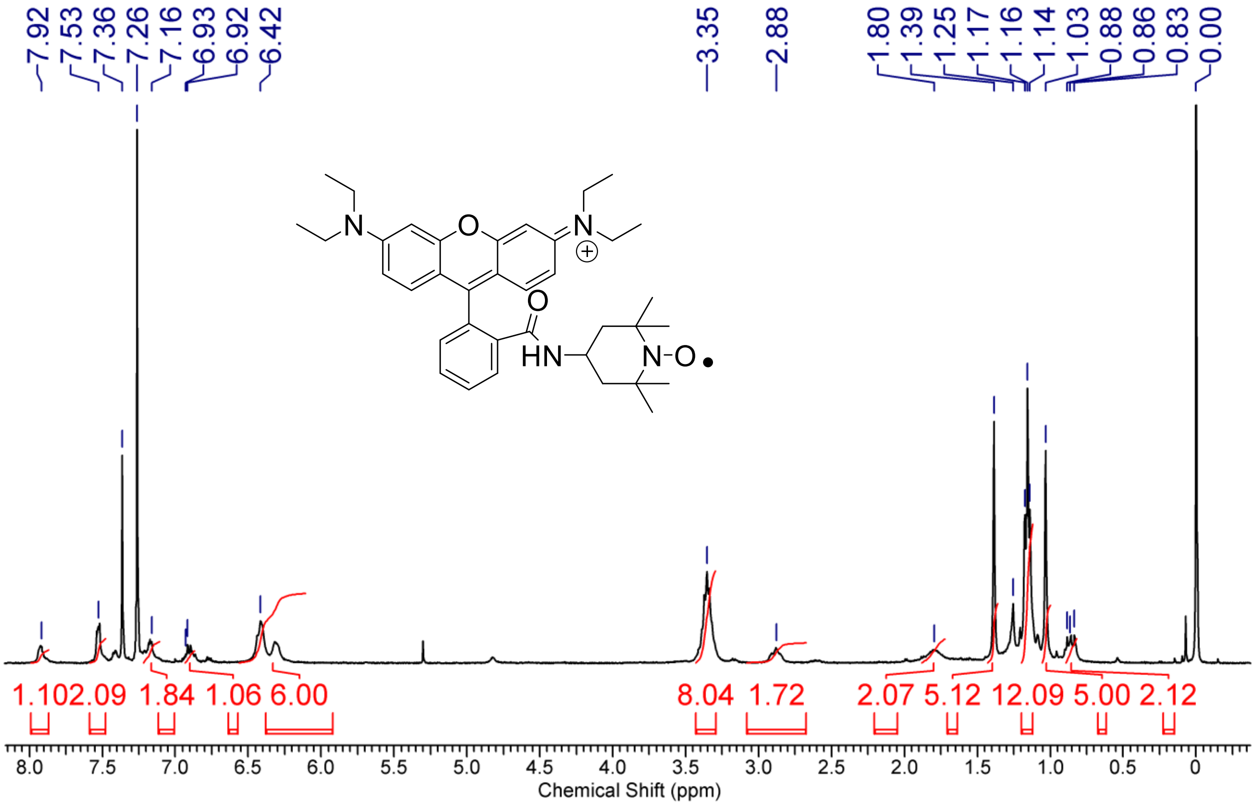

Figure S1. ^{1}H NMR of compound **RB-TEMPO** (400 MHz, $CDCl_3$), 25°C.

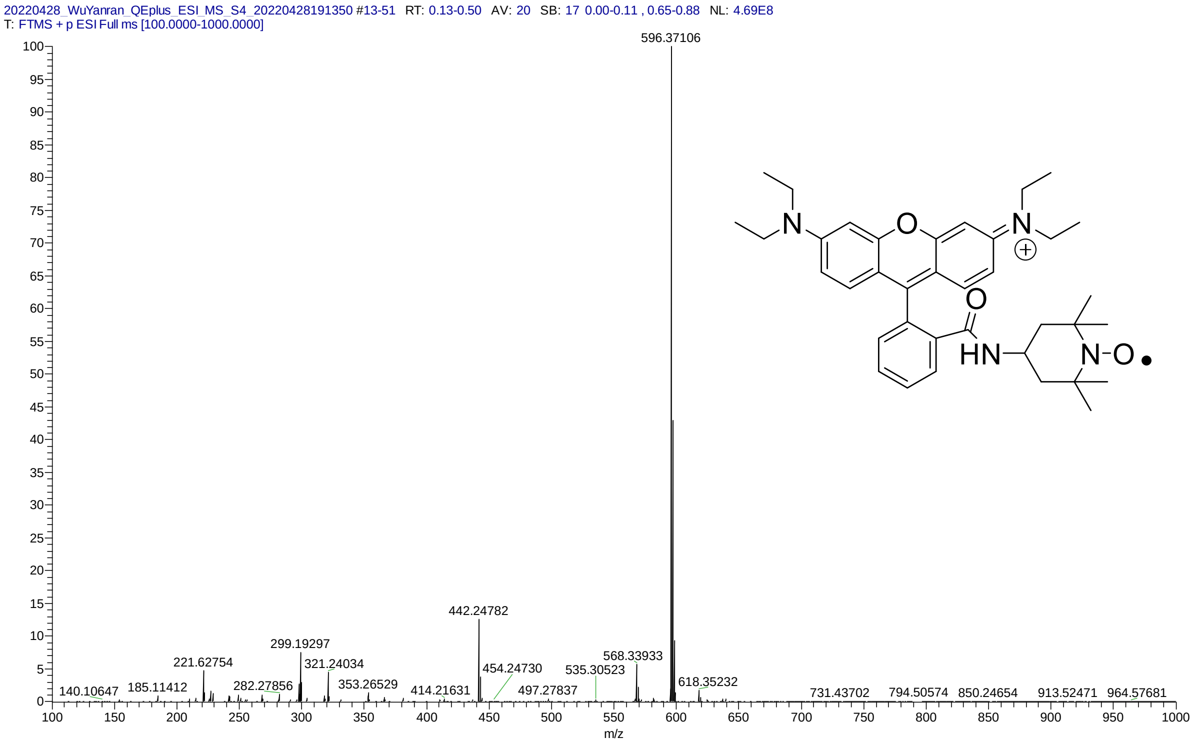

Figure S2. MALDI HR MS of compound **RB-TEMPO**. 25 °C.

3. UV–vis Absorption Spectra

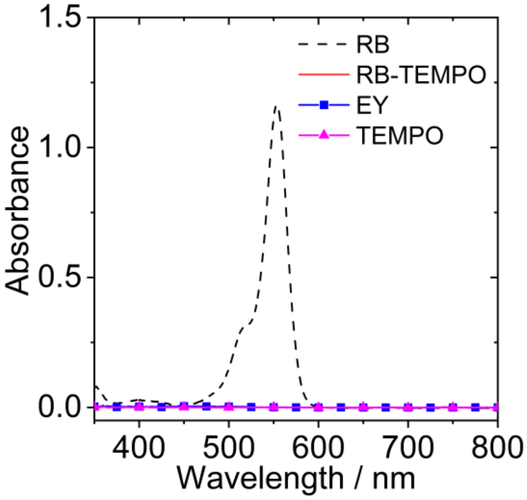

Figure S3. UV–vis absorption spectra of the compounds in dichloromethane (DCM), c = 1.0×10^{-5} M, 3mL, 20 °C.

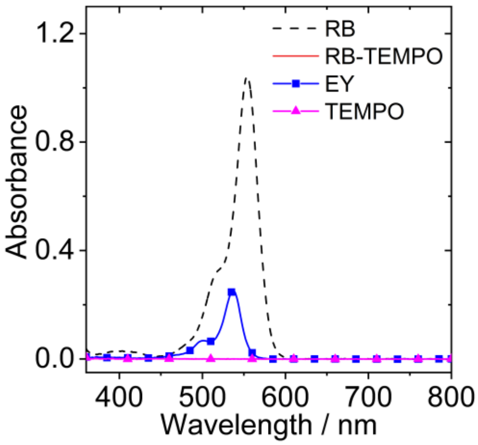

Figure S4. UV–vis absorption spectra of the compounds in acetonitrile (ACN), $c = 1.0 \times 10^{-5}$ M, 3mL, 20 °C.

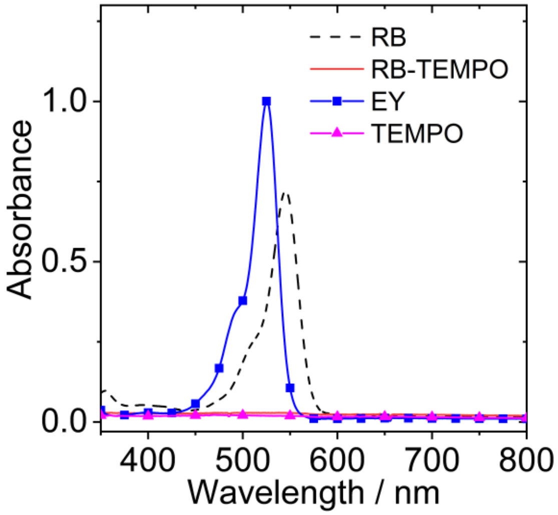

Figure S5. UV–vis absorption spectra of the compounds in methanol (MeOH), $c = 1.0 \times 10^{-5}$ M, 3mL, 20 °C.

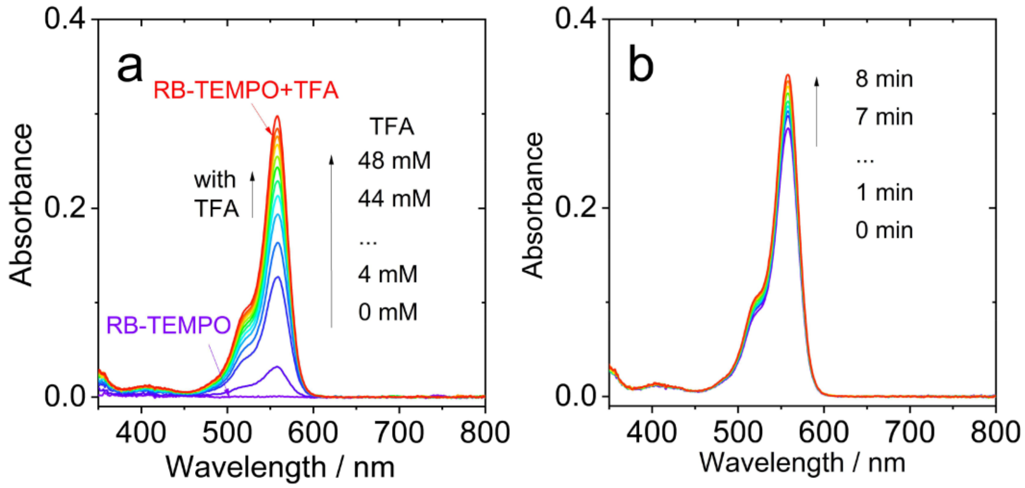

Figure S6. UV–Vis absorption spectra change with (a) the amount of trifluoroacetic acid added to **RB-TEMPO** solution and (b) reaction time after adding 48 mM trifluoroacetic acid to **RB-TEMPO** solution in DCM, $c = 1.0 \times 10^{-5}$ M, 3 mL, 20 °C.

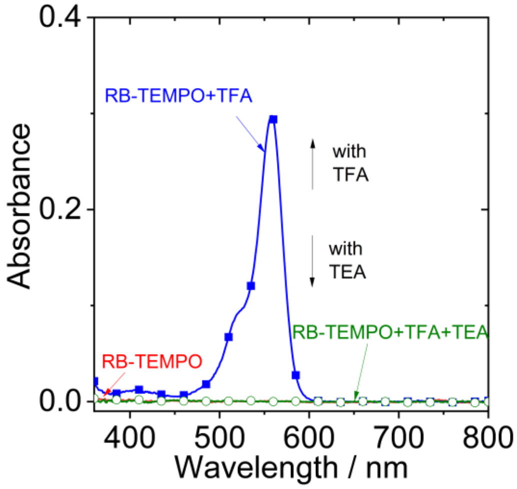

Figure S7. UV–vis absorption spectra of **RB-TEMPO** with the addition of TFA (48 mM) or trimethylamine (TEA, 280 mM) in DCM. $c = 1.0 \times 10^{-5}$ M, 3 mL, 20 °C.

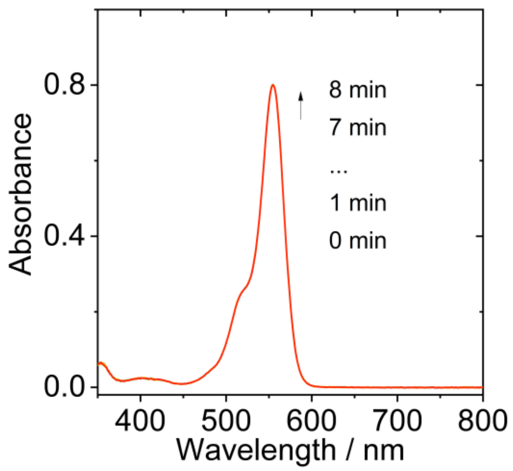

Figure S8. UV–Vis absorption spectra change with reaction time after adding 48 mM trifluoroacetic acid to **RB-TEMPO** solution in EtOH. $c = 1.0 \times 10^{-5}$ M, 3 mL, 20 °C.

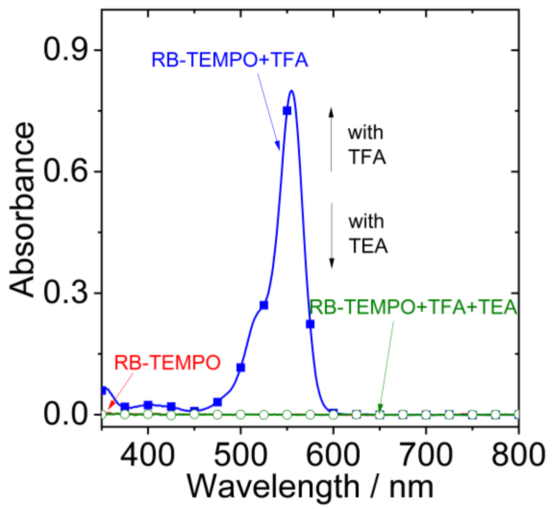

Figure S9. UV–vis absorption spectra of **RB-TEMPO** with the addition of TFA (48 mM) or trimethylamine (TEA, 280 mM) in EtOH. $c = 1.0 \times 10^{-5}$ M, 3 mL, 20 °C.

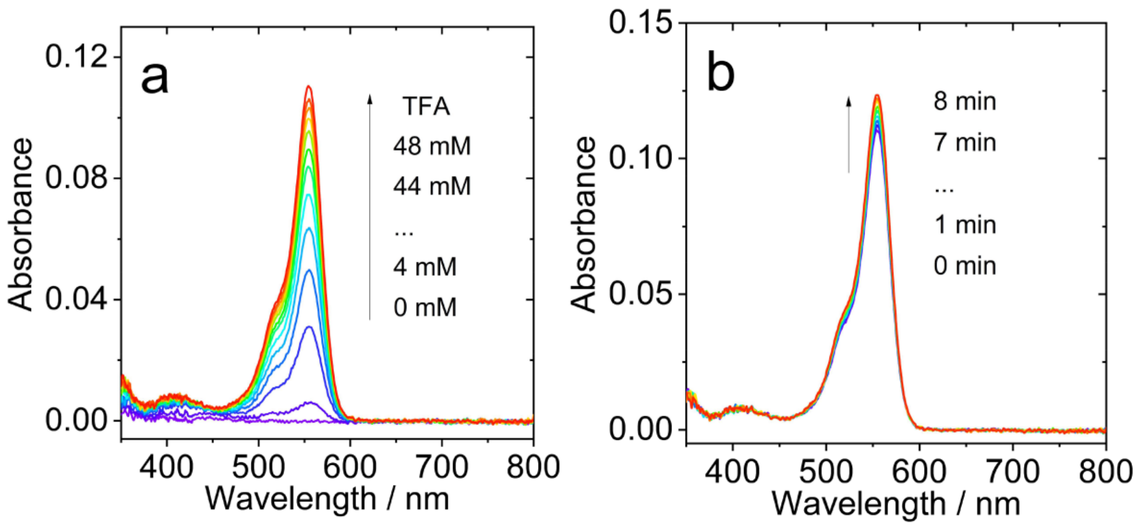

Figure S10. UV–Vis absorption spectra change with (a) the amount of trifluoroacetic acid added to **RB-TEMPO** solution and (b) reaction time after adding 48 mM trifluoroacetic acid to **RB-TEMPO** solution in ACN. $c = 1.0 \times 10^{-5}$ M, 3 mL, 20 °C.

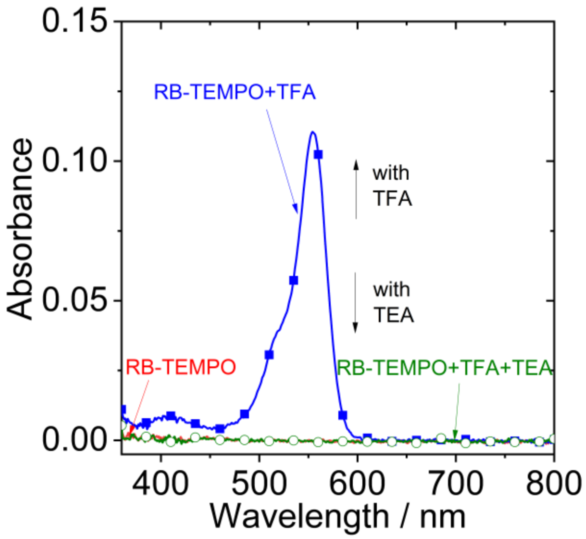

Figure S11. UV–vis absorption spectra of **RB-TEMPO** with the addition of TFA (48 mM) or trimethylamine (TEA, 280 mM) in ACN. $c = 1.0 \times 10^{-5}$ M, 3 mL, 20 °C.

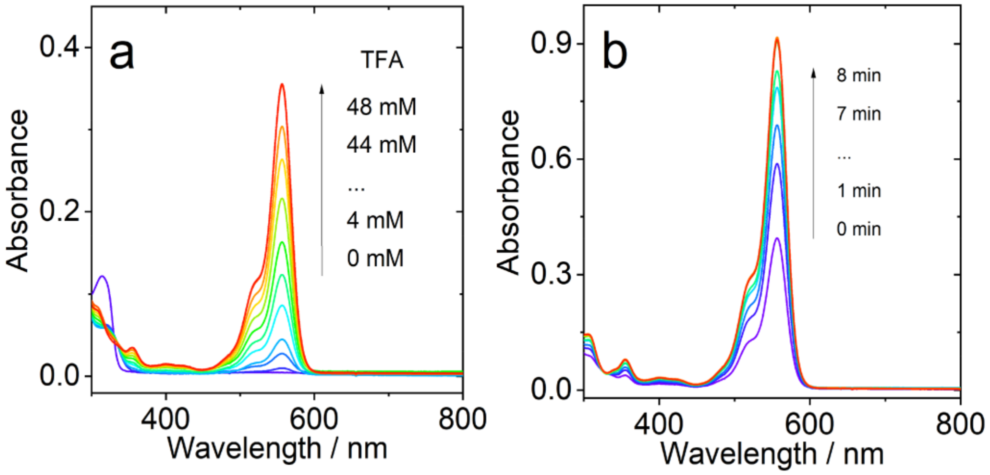

Figure S12. UV–Vis absorption spectra change with (a) the amount of trifluoroacetic acid added to **RB-TEMPO** solution and (b) reaction time after adding 48 mM trifluoroacetic acid to **RB-TEMPO** solution in MeOH. $c = 1.0 \times 10^{-5}$ M, 3 mL, 20 °C.

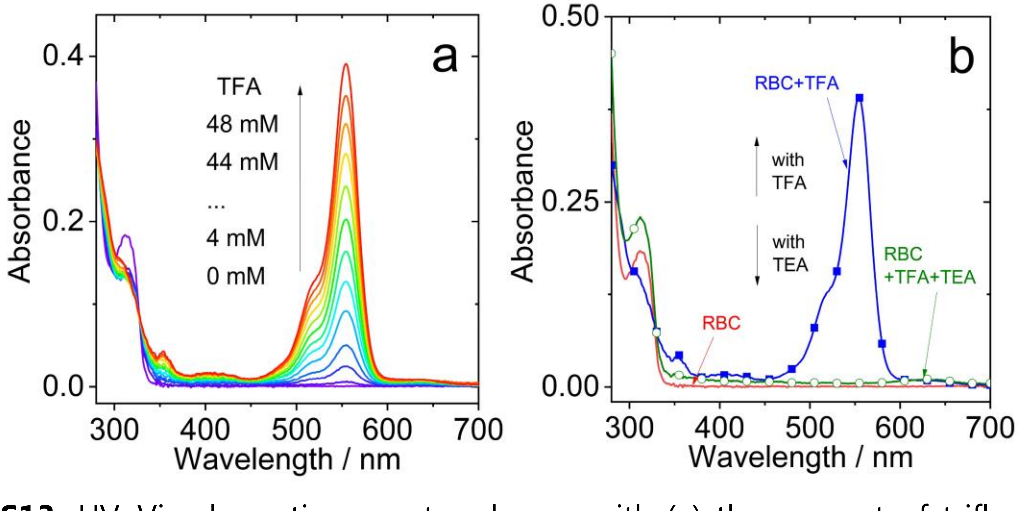

Figure S13. UV–Vis absorption spectra change with (a) the amount of trifluoroacetic acid added to **RBC** solution and (b) with the addition of TFA (48 mM) or trimethylamine (TEA, 280 mM) in EtOH. $c = 1.0 \times 10^{-5}$ M, 3 mL, 20 °C.

4. Fluorescence Spectra

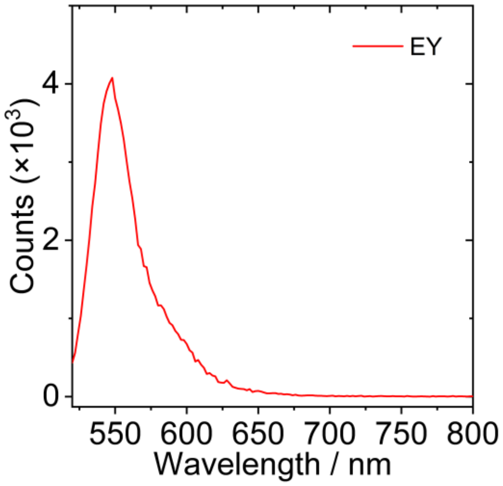

Figure S14. Fluorescence emission spectra of **EY** in EtOH. λ_{ex} = 510 nm. c = 1.0 × 10^{-5} M. 20 °C.

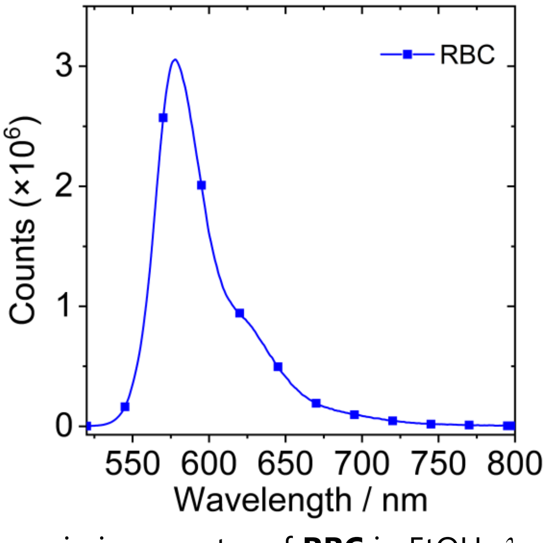

Figure S15. Fluorescence emission spectra of **RBC** in EtOH. λ_{ex} = 510 nm. c = 1.0 × 10^{-5} M. 20 °C.

5. Fluorescence Lifetime Spectra

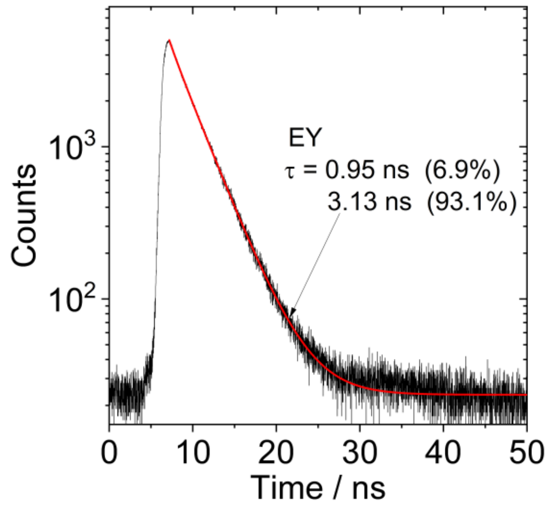

Figure S16. Fluorescence decay traces of **EY** (λ_{ex} = 510 nm) in EtOH, λ_{em} = 548 nm, c = 1.0 × 10^{-5} M, 20 °C.

6. Singlet Oxygen Quantum Yields

The singlet oxygen ($^1O_2$) quantum yields (Φ_Δ) were measured by using 1,3-Diphenylisobenzofuran (DPBF) as a $^1O_2$ scavenging agent [1], the irradiation time-dependent absorption change was monitored with UV-Vis absorption spectrophotometer. **RB**, **RB-TEMPO** and **EY** were excited with 540 nm (methylene blue was used as standard compound, Φ_Δ=57% in dichloromethane). DPBF and target photosensitizer were mixed in 3 mL ethanol, and the absorbance of DPBF at 416 nm was adjusted to be close to 1.0, target photosensitizer' s absorbance at the excitation wavelength was 0.2 – 0.3, the solutions were excited with a 300 W Xenon lamp (pulsed laser, λ_{ex} = 540 nm), the linear relationship between the irradiation time and the absorbance at 416 nm wavelength was recorded every 15 s, and the slope of the curves

was calculated. Singlet oxygen quantum yields were calculated by using the following equation:

$$\Phi_{\Delta} = \Phi_{\Delta,\mathrm{std}}\left(\frac{1-10^{-A_{\mathrm{std}}}}{1-10^{-A_{\mathrm{sam}}}}\right)\left(\frac{\mathrm{m_{sam}}}{\mathrm{m_{std}}}\right)\left(\frac{\eta_{\mathrm{std}}}{\eta_{\mathrm{sam}}}\right) \tag{1.}$$

Where 'sam' and 'std' designate the target compounds and standard compounds respectively. 'A' stands for the absorbance, 'm' is the slope of the curves of the absorbance of DPBF at 416 nm versus the irradiation time, and 'η' is the refractive index of the solvents.

Table S1. The experimental results for time-depended absorbance change of DPBF in ethanol upon irradiation with a 300W Xenon lamp at 540 nm (light intensity: 3.0 W/m^2) in the presence of **MB**/**EY**/**RB-TEMPO**.

Compounds	0 s	15 s	30 s	45 s	60 s	75 s	90 s	105 s
**MB**	1.611	1.555	1.498	1.444	1.391	1.332	1.271	1.209
**EY**	1.559	1.533	1.503	1.475	1.443	1.411	1.378	1.352
**RB-TEMPO**	0.754	0.702	0.650	0.604	0.559	0.508	–	–

7. Nanosecond Transient Absorption Spectra

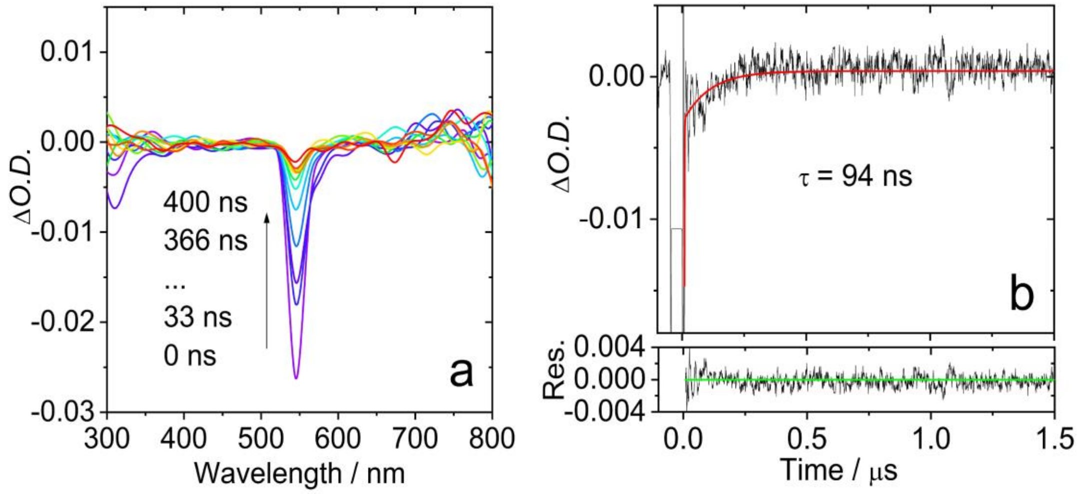

Figure S17. (a) Nanosecond transient absorption spectra of **RB** upon pulsed laser excitation (λ_{ex} = 550 nm). (b) Decay trace of **RB** at 545 nm, c = 1.0 × 10^{-5} M, 3 mL in deaerated methanol, 20 °C

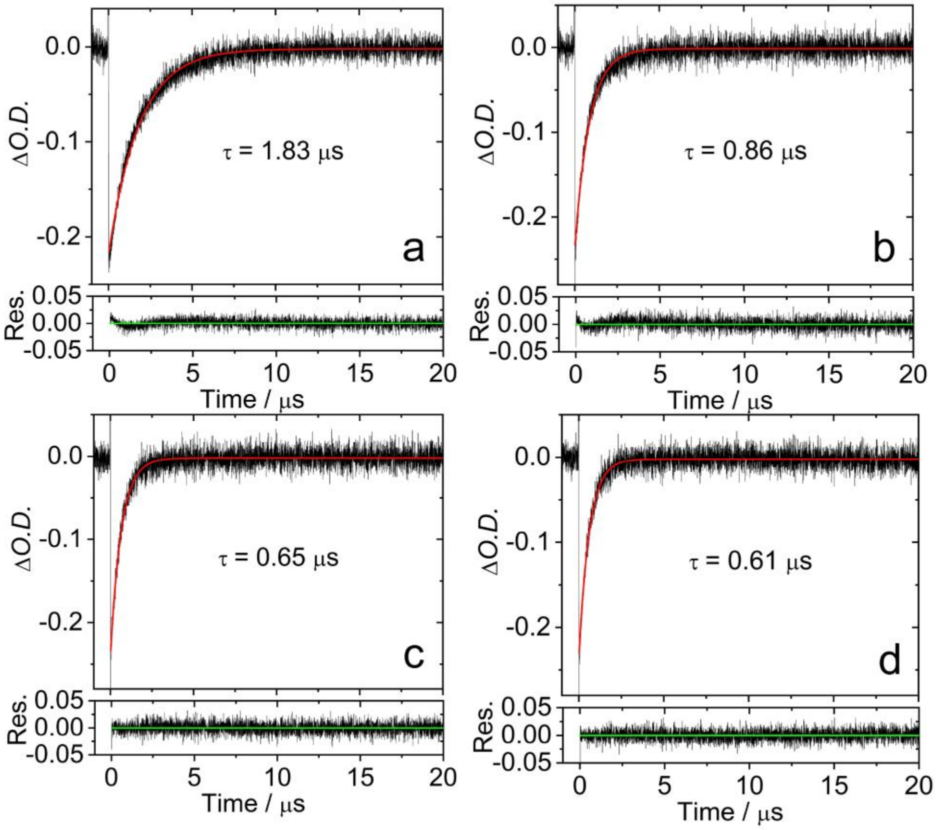

Figure S18. Triplet lifetime after adding (a) 5 equivalent, (b) 10 equivalent, (c) 20 equivalent and (d) 30 equivalent **TEMPO** to **EY** solution at 525 nm in deaerated methanol (**TEMPO** radical quenched triplet lifetime), c = 1.0 × 10^{-5} M, 20 °C.

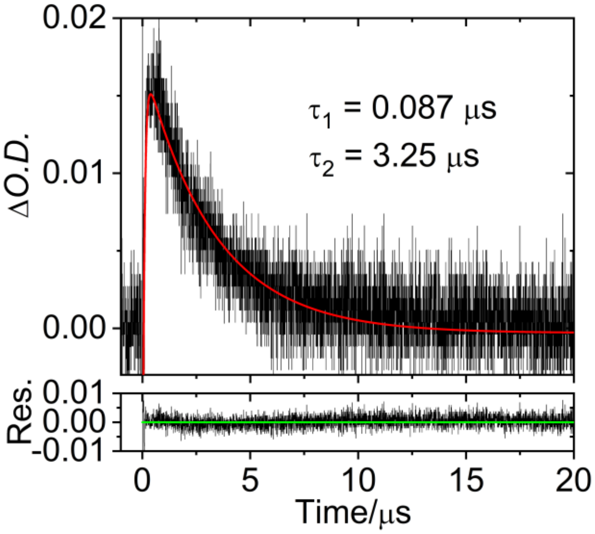

Figure S19. Decay trace of **EY** at 600 nm, c = 1.0 × 10^{-5} M, 3 mL in deaerated methanol, 20 °C

8. Photopolymerization and photobleaching

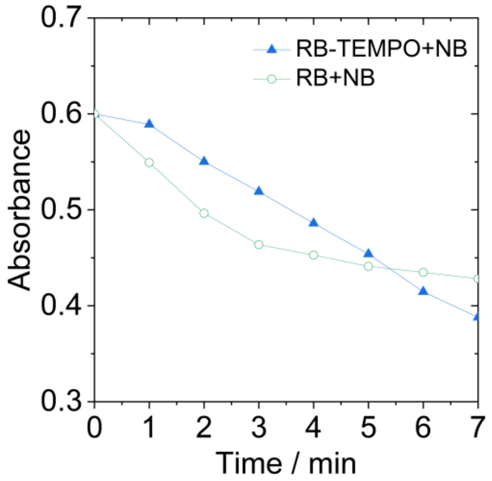

Figure S20. Photobleaching of the compounds in the presence of electron donor upon white light irradiation, monitored by the evolution of the absorbance of **RB-TEMPO**/NB and **RB**/NB at 555nm vs. irradiation time upon exposure to a 35W Xeon lamp (unfiltered white light intensity: 80 mW/cm^2) in deaerated acetonitrile. c[**RB**] = 1.0 × 10^{-5} M, c[**RB-TEMPO**] = 1.0 × 10^{-5} M, c[NB] = 1.0 × 10^{-3} M, 20 °C.

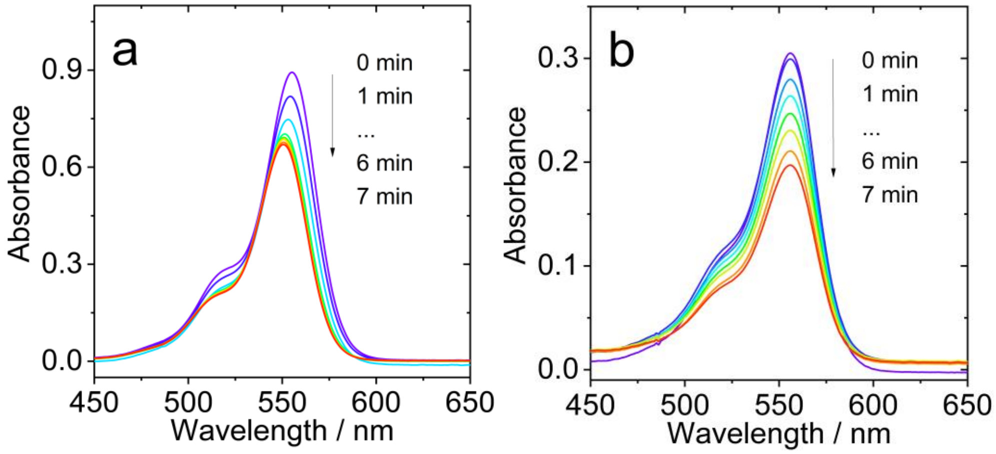

Figure S21. Photobleaching of the compounds in the presence of electron donor NB upon white light irradiation (unfiltered white light intensity: 80 mW/cm^2) in deaerated acetonitrile. (a) **RB**/NB, and (b) **RB-TEMPO**/NB, every absorption spectrum were recorded after 1 min. of consecutive photoirradiation. c[**RB**] = 1.0 × 10^{-5} M, c[**RB-TEMPO**] = 1.0 × 10^{-5} M, c[NB] = 1.0 × 10^{-3} M, 20 °C.

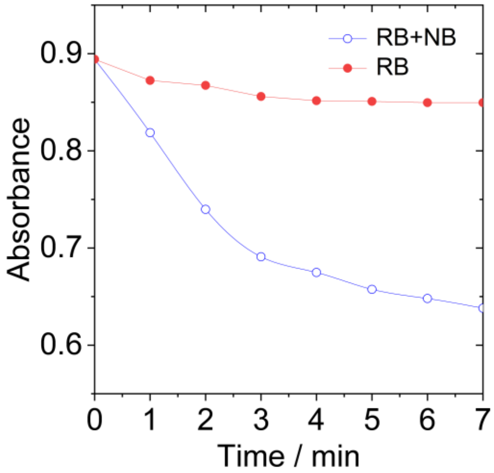

Figure S22. The evolution of the absorbance of **RB** and **RB**/NB at 555nm after 1 min of irradiation each time in deaerated acetonitrile (unfiltered white light intensity: 80 mW/cm^2), c[**RB**] = 1.0 × 10^{-5} M, c[NB] = 1.0 × 10^{-3} M, 20 °C.

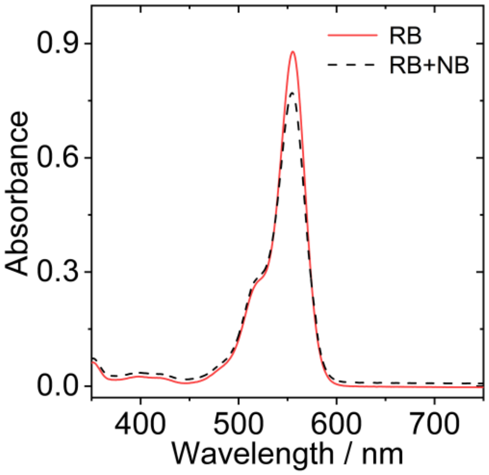

Figure S23. UV–vis absorption spectra of **RB** and **RB**/NB mixture in deaerated acetonitrile. c[RB] = 1.0 × 10^{-5} M, c[NB] = 1.0 × 10^{-3} M, 20 °C.

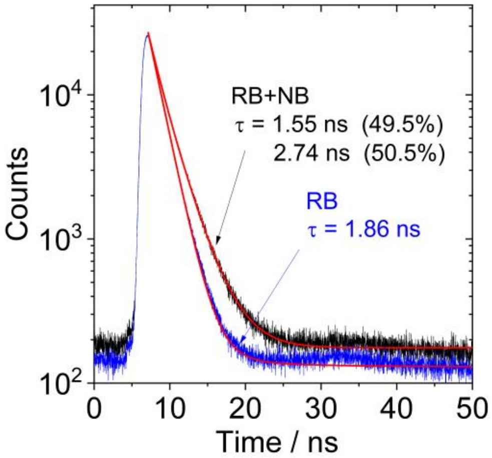

Figure S24. Fluorescence decay traces of **RB** and **RB**/NB in deaerated acetonitrile, λ_{em} = 570nm, λ_{ex} = 510 nm, c[**RB**] = 1.0 × 10^{-5} M, c[NB] = 1.0 × 10^{-3} M, 20 °C.

9. Electrochemical Study

Table S2. Redox Potential, Electron transfer Free Energy (ΔG)

Compounds	E_{RED}[a] / V	E_{ox}[a] / V	E_{00} / eV	ΔG / eV
**RB**	-1.15/<br>-1.37	+0.85/<br>+0.97	1.72[b]/<br>1.72[c]	-0.14[d]/<br>-0.14[e]
**NB**	-[f]	+0.43	-[g]	-[g]

[a] The value is obtained by setting the oxidation wave of Fc+/Fc as 0. [b] The singlet energy level of Rhodamine B. [c] The triplet energy level of Rhodamine B. [d] Singlet electron transfer of RB/NB. [e] Triplet electron transfer of RB/NB. [f] Not observed. [g] Not applicable.

10. References

[1] T. Entradas, S. Waldron and M. Volk, The detection sensitivity of commonly used singlet oxygen probes in aqueous environments, *J. Photochem. Photobiol. B, Biol.*, 2020, **204**, 111787.
